# “Ferula assa-foetida L” or “Foeniculum vulgare”? Which one is more effective in the management of polycystic ovarian syndrome? A randomized, placebo controlled, triple-blinded

**DOI:** 10.22038/AJP.2022.21389

**Published:** 2023

**Authors:** Fatemeh Ghavi, Fatemeh Shakeri, Somayeh Abdolahian

**Affiliations:** 1 *Department of Midwifery, School of Nursing and Midwifery, Shiraz University of Medical Sciences, Fars, Iran*; 2 * Department of Midwifery, School of Nursing and Midwifery, Jahrom University of Medical Sciences, Fars, Iran*; 3 *Department of Midwifery and Reproductive Health, School of Nursing and Midwifery, Shahid Beheshti University of Medical Sciences, Tehran, Iran*

**Keywords:** Ferula, Ferula assa-foetida L Fennel, Foeniculum vulgare Polycystic ovarian syndrome PCOS

## Abstract

**Objective:**

There are few evaluation about the effects of Fennel and Ferula on human polycystic ovary syndrome (PCOS). The goals of this study were to evaluate and compare the effectiveness of two medicinal plants of the Apiaceae family (Fennel and Ferula) in management of PCOS.

**Materials and Methods:**

The sample size was 47 participants with PCOS who were randomly divided into 3 groups. The Ferula assa-foetida L group received 100 mg of Ferula (n=14), the Foeniculum vulgare group received 46 mg of Fennel (n=15), and the placebo group received placebo twice daily for 3 months (n=14).

**Results:**

Before the intervention, there were no significant differences between groups in terms of clinical parameters, endometrial thickness, or ovarian volume. After the interventions, the number of ovarian follicles was decreased in the Ferula and Fennel groups as compared to the placebo group (p<0.05). The number of ovarian follicles in both ovaries in the Ferula and Fennel group decreased and this decrease was significant in the right side as compared to placebo group. Our findings showed significant changes in dehydroepiandrosterone sulfate (DEHAS) and thyroid-stimulating hormone (TSH) levels after the intervention (p<0.03) between the Ferula and Placebo groups.

**Conclusion:**

Since use of Ferula could make significant changes in TSH and DEHAS levels and decrease the number of right and left ovarian follicles compared to Fennel and placebo, it can be concluded that this herbal medicine is more effective than Fennel in managing PCOS.

## Introduction

Polycystic ovarian syndrome (PCOS) which is usually caused by hormonal imbalances, has effects on childbearing years of women. Androgen imbalances can increase the prevalence of several disorders including insulin resistance, diabetes, hyperlipidemia, and cardiovascular diseases (Zeng et al., 2020). Additionally, these disorders can cause problems such as hirsutism, acne, irregular menstrual cycle and infertility (Jalilian et al., 2015). Different treatments are suggested for PCOS, like lifestyle modification (Abdolahian et al., 2020), drug therapy (Lashen, 2010) and herbal medicine (Kwon et al., 2018).

In Iran, gynecological disorders such as dysmenorrhea (Jahangirifar et al., 2018) and oligomenorrhea (Akha et al., 2014) are traditionally treated by herbal medicines like *Ferula assa-foetida *L (Ferula that is locally referred to as “*Anghuzeh*”) or *Foeniculum vulgare *(Fennel that is locally referred as Razianeh) (Khazdair and Boskabady, 2015; Badgujar et al., 2014, Iranshahy and Iranshahi, 2011). Both of these drugs have antihirsutism, antibacterial, anthelmintic, and anticancer effects and are traditionally used for treatment of variousproblems: dysmenorrhea, cancer, and colic in children (Zare et al., 2011) and vaginal infection (Bagherinasab et al., 2019). These herbal medicines belongs to the Umbelliferae (Apiaceae) family (Sahebkar and Iranshahi, 2010). The biochemical activities of the ferutinin in the Umbelliferae family can modulate estrogen signaling like phytoestrogens (Ayoubi et al., 2013; Bagheri et al., 2015). Ferutinin as an elective Estrogen Receptor Modulator (SERM) regulates Estrogen receptor alpha (ERα) and Estrogen Receptor Beta (ERβ) (Al-Ja’fari et al., 2011). PCOS is a disorder primarily signalized by signs and symptoms of androgen excess (Ndefo et al., 2013), and the consequence of Ferula injection in male rats showed a decrease of testosterone hormones. Also, injection of Fennel in rats with PCOS had increasing effects on serum concentration of follicle- stimulating hormone (FSH), and decreasing effects on Luteinizing hormone (LH) and testosterone (Karampoor et al., 2014).

To our knowledge, there are few evaluation about the effects of Ferula and Fennel on human PCOS. The goals of this study were to evaluate and compared the effectiveness of two medicinal plants of the Apiaceae family (Fennel and Ferula) in management of PCOS.

## Materials and Methods

Study design

This controlled clinical trial was a triple-blinded study in which, 47 participants with PCOS were randomly entered into 3 groups (One placebo and two intervention groups).

Participants

The young girl students (18-30 years old) diagnosed with PCOS supported Rotterdam criteria (Table 1) were selected as participants. Exclusion criteria were as follows: Medical conditions like androgen- secreting tumors, hyper prolactinoma, thyroid disorders, Cushing’s syndrome (determined by an acceptable laboratory assay), pregnancy or lactation.

**Table 1:** Rotterdam diagnostic criteria for PCOS

**Rotterdam (2003) Diagnostic criteria for PCOS - two out of three of:**
Clinical Hyperandrogenism (Ferriman-Gallwey Score >8) or Biochemical Hyperandrogenism (Elevated Total/Free Testosterone)
oligomenorrhea (Less Than 6-9 Menses per Year) or Oligo-Ovulation
Polycystic Ovaries on Ultrasound (>= 12 Antral Follicles in One Ovary or Ovarian Volume >= 10 cm3)

Sample size

For calculation of sample size, we used a formula which was presented by another article 981. Then, subtracted essence was formed into pearl-shaped soft capsules (100 mg). The major constituents of asafoetida are the resin (40–64%), gum (25%) and essential oil (10–17%). Ferula toxicity and safety were evaluated by a systematic.

A significance level of 0.05, a power level of 0.80 with an attrition rate (10%) were chosen for this study and according to the formula, 18 samples should enroll to the trial; 15-17 samples were included in each group.

Random allocation

Simple random allocation was accomplished by a random Table. In this step, each participant was assigned randomly to one of the three groups.

In concealment step, packages containing herbal medicines or placebo had identical shape and size with a novel code assigned to each package. Only pharmacist knew that each code belonged to which group.

Blinding

The participants and researchers were not aware of the contents of each package. After 3 months of interventions, the contents of the packages and capsules were disclosed by the pharmacist for statistical analysis. S.A created the random sequence and data analysis, F. GH assessed and registered the samples according to inclusion and exclusion criteria, F. SH assigned the samples to the groups.

Preparation of capsules

Medicinal plant market (Tabib Daru), Kashan, Iran was the supplier of the Ferula root and stem. *Ferula assa foetida *L essence was produced by distilling the root and stem in the department of phytopharmaceuticals (Traditional Pharmacy) (School of Pharmacy- Shiraz University of Medical Sciences- Shiraz- Iran) with Herbarium Voucher Number review study which showed only skin allergic sign in infants (Zare AR *et al.*, 2011).

The substantial components of fennel essential oils included 50-80% Trans- Anethole, 20-30% Fenchone and 1-5% Estragole (Methylchavicol). Fennel soft capsules were provided by the Barij Essence Drug Company (Mashhad Ardehal- Kashan- Iran). (http://barijessence.com/en/product/fenneli n-softcap/) *Foeniculum vulgare *essence was produced by distilling the fennel seeds with water vapor and then, subtracted essence was formed into pearl-shaped soft capsules (46 mg). Fennel safety had been reported by another study (Badgujar *et al.*, 2014).

The placebo (pearl-shaped soft capsules) contained starch of 100 mg soy and was produced by the Barij Essence Drug Company. In order to blinding, each capsule in 3 groups had comparable shape, color and size.

Every participant in the groups received capsules twice daily for 3 months.

Data collection

The primary outcomes were clinical parameters (such as body mass index (BMI), modified Ferriman-Gallwey (mFG) score, and menstrual periods), which were measured at the start and end of the trial. MFG scale was used for assessing the hirsutism score in these sites: upper lip, chin and sides of the face. This scale has five grades, and the scores range from zero to four for each site (Amiri *et al.*, 2017). BMI was measured as follows: BMI (kg/m^2^) = weight (kg) / height^2^ (m^2^). Duration of menstruation cycles was measured from the first day of one menstrual cycle to the first day of the next cycle according to participants’ reports.

Hormonal parameters (such as DHEAS, TSH, and free testosterone (FT), Follicle stimulating hormone (FSH), luteinizing hormone (LH), and prolactin (PRL)), and ultrasound parameters (such as endometrial thickness, ovarian volume and number of follicles in both ovaries) were secondary outcomes. A blood sample was derived from each participant; then, its plasma was separated and kept in -20ºC until it was used by DHEAS, FT, FSH, and LH immunoassay kits (Monobind Inc., CA, US) based on the manufacturer’s instructions. Testosterone, PRL, and TSH were measured by using the enzyme- linked immunosorbent assay (Monobind Inc., Germany). Trans abdominal ultrasound by one expert sonographer was done for all participants (except participants with oligo or amenorrhea) in the follicular phases of the menstrual cycle for determination of ovarian volumes, number of follicles of both ovaries, and endometrial thickness at 4.4-MHz (Alpinion, Seoul, South Korea).

 Ethics consideration

Ethics considerations of this study were approved by Ethics and Research Committee of Jahrom University of Medical Sciences- Jahrom- Iran (reference number: ums.REC.1393.023). Also the protocol of this trial was submitted in the Iranian Randomized Clinical Trial (IRCT2016040427207N1).

Statistical analysis

All statistical analysis was accomplished by using IBM SPSS Statistics version 14. Quantitative data are described as mean and standard deviation with 95% confidence interval. The Kolmogorov–Smirnov test was used for assessment of normal distribution of data, and a non-parametric test was conducted to evaluate the parameters. To analyze before and after intervention means for each variable, a Wilcoxon and ANCOVA test was used.

**Figure 1:**
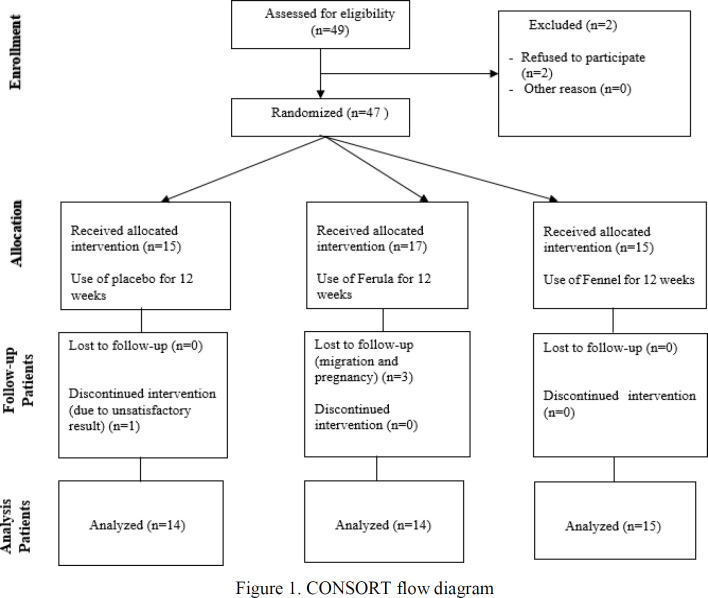
CONSORT flow diagram

## Results

Of the 47 participants entered to the current trial, 4 subjects failed to finish the trial and were excluded from the analysis, therefore, after 3 months of the intervention, 43 students finalized the study (Figure 1). There were insignificant differences in mean age between the groups (The mean age of Ferula group: 22.93±1.5 years, Fennel group: 23.60±2.32 years, and placebo group: 23.14±2.7 years). Before and after the intervention, no significant changes were observed between the three groups in clinical parameters (BMI, mFG score, or menstruation duration) (Table 2).

The result showed that DHEAS (p=0.03) and TSH (p=0.004), significantly changed in the Ferula group vs. placebo. Hormonal parameters (FSH, LH, prolactin, and free testosterone) before and after intervention were not significantly different between the three groups (Table 3).

**Table 2:** Comparison of clinical parameters in patients treated with Ferula, Fennel, and placebo before and after the intervention

clinical parameters	Group	Mean±SD (95%CI) Before intervention	Mean±SD (95%CI) After intervention	p. value*
	Fennel	23.50±5.03	22.97±4.44	0.290
	Ferula	23.8±1.1	23.37±4.25	0.117
BMI (kg/m2)	placebo	25.3±1.2	25.24±4.6	0.859
	p. value**	0.261		
	Fennel	9.8±2.54	7.2±3.67	0.05
Ferriman-Gallwey Score	Ferula	7.21±6.81	3.92±4.23	0.02
	placebo	6.57±4.9	6.30±4.23	0.918
	p. value**	0.148		
	Fennel	45.40±19.00	40.33±15.55	0.35
Menstruation cycle length (days)	Ferula	40.29±18.63	39.50±23.65	0.83
	placebo	49.36±19.62	39.14±11.61	0.56
	p. value**	0.342		

*Wilcoxon

** Kruskal–Wallis

**Table 3:** Comparison of hormonal parameters in patients treated with Ferula, Fennel, and placebo before and after the intervention

Hormone parameter	Group	Mean±SD (95%CI) Before intervention	Mean±SD (95%CI) After intervention	p. value*
Dehydroepiandrosterone sulfate (g/ml)	Ferula	2.28±0.29	1.51±0.865	0.005
	Fennel	2.94±0.82	7.52±19.48	0.158
	placebo	2.38±0.24	2.96±1.01	0.490
	p. value**	0.667	0.03	
Free Testosterone (pg/ml)	Ferula	3.7±0.84	1.52±1.615	0.02
	Fennel	2.55±1.77	1.76±1.39	0.136
	placebo	3.8±0.6	2.06±1.5	0.019
	p. value**	0.454	0.376	
Follicle-stimulating hormone (mlU/ml)	Ferula	4.11±0.27	3.742±2.53	0.221
	Fennel	5.63±2.20	5.06±2.39	0.232
	placebo	5.34±0.55	3.91±1.34	0.07
	p. value**	0.50	0.227	
Luteinizing hormone (mlU/ml)	Ferula	10.96±2.34	11.1±6.14	0.639
	Fennel	10.93±5.83	8.76±3.93	0.158
	placebo	7.64±0.55	7.57±4.5	0.925
	p. value**	0.511	0.104	
Thyroid-stimulating hormone (mlU/ml)	Ferula	1.93±0.20	1.25±0.43	0.013
	Fennel	2.01±1.33	2.09±0.90	0.124
	placebo	1.57±0.23	1.92±0.72	0.583
	p. value**	0.05	0.004	
Prolactin (g/ml)	Ferula	17.65±1.98	14.25±8.35	0.109
	Fennel	18.07±11.74	20.51±14.33	0.754
	placebo	19.69±1.93	19.05±14.7	0.594
	p. value**	0.376	0.178	

*Wilcoxon

** Kruskal–Wallis

Both ovarian volume and endometrial thickness were not significantly different after the intervention in the Ferula and Fennel groups compared to the placebo group (Table 4).

The results showed the number of follicles in the right and left ovaries (p<0.01) significantly changed in the Ferula and Fennel groups vs. placebo group (Table 5).

The number of follicles within the right ovary (p<0.0001) in Ferula group significantly decreased.

**Table 4:** Comparison of ultrasound parameters in patients treated with Ferula, Fennel, and placebo before and after the intervention

Trans abdominal ultrasound	Group	Mean±SD (95%CI) Before intervention	Mean±SD (95%CI) After intervention	p. value*
Endometrial thickness	Ferula	6.79±0.74	6.28±2.43	0.573
	Fennel	6.23±1.50	5.81±1.74	0.414
	placebo	5.46±0.44	5.82±1.81	0.609
	p. value**	0.178	0.667	
right ovary volume	Ferula	11.31±1.75	9.42±5.8	0.64
	Fennel	10.30±2.63	8.17±3.32	1.000
	placebo	9.04±0.87	9.91±3.58	0.039
	p. value**	0.511	0.511	
left ovary volume	Ferula	10.18±1.73	11.11±6.95	0.505
	Fennel	10.67±3.02	8.26±3.06	0.06
	placebo	8.73±0.61	8.88±3.08	0.655
	p. value**	0.946	0.603	

*Wilcoxon

** Kruskal–Wallis

**Table 5:** Comparison of the number of follicles in right and left ovary in patients treated with Ferula, Fennel, and placebo before and after the intervention

Left ovarian follicular number					Right ovarian follicular number			
	Mean difference (SE)	95% Confidence Interval for Difference b		p*	Mean difference (SE)	95% Confidence Interval for Difference b		p*
		Lower bound	Upper bound			Lower bound	Upper bound	
Fennel vs. placebo	-2.425	-4.241	-0.608	0.01	-2.29	-3.780	-0.810	0.003
Ferula vs. placebo	-4.079	-5.72	-2.43	0.000	-5.131	-6.444	-3.819	0.00
Ferula vs. Fennel	-1.65	-3.40	0.097	0.063	-2.83	-4.204	-1.469	0.00

*ANCOVA. Based on estimated marginal means*. The mean difference is significant at 0.05 level

## Discussion

This study was conducted to assess the effects of Ferula and Fennel in PCOS management. Before the intervention, there were no significant differences in terms of clinical parameters, endometrial thickness, and ovarian volume among the groups. After the interventions, a reduction in the number of ovarian follicles was reported in the Ferula and Fennel groups as compared with the placebo group (p<0.05). The number of ovarian follicles in both ovaries in Ferula and Fennel group decreased and this decrease was significant in the right-side ovary as compared to placebo group.

Significant differences in DEHAS and TSH levels were found after the intervention (p<0.03) between the Ferula and Placebo groups.

Since the herbal medicines used in this study were both anti-obesity and anti-fat, their effectiveness on BMI was evaluated and the results showed that BMI in the three groups was not significantly changed before and after the intervention. Similarly, it was shown Fennel has no effect on weight loss in women unless it is used combined with a diet (Hosseini Marnani et al., 2020; Saghafi et al., 2017). PCOS international guidelines recommend a six-month duration as an optimal intervention duration to reduce BMI in PCOS women (Teede et al., 2018). One study has found that a Fennel seed can help to 1.5 unit decrease in BMI after 6 months (Mokaberinejad et al., 2019)

This present study results showed that the mFG score mean in three groups did not significantly change after interventions. The topical cream consumption (1% and 2% of fennel) for 12 weeks decreased idiopathic hirsutism (Javidnia et al., 2003) therefore, it might be concluded that the topical use of these herbal medicines on the skin is more effective than their oral consumption. Also use of combination of Fennel and black cumin For 4 months has been positive change in FG score in PCOS patients (Mohammadi et al., 2020).

Our results showed that the menstrual duration in PCOS women after 3 months of interventions had not any significant change between Ferula and fennel group as compared to placebo group. Mokaberinejad et al study showed the use of Fennel seed for 3 months was not effect on mensturtion lengh but after 6 months, it has been effective for oligomenorrhea treatment (Mokaberinejad et al., 2019). It was reported that phytoestrogens were not related to cycle length but may be associated with menstrual regularity (Levine et al., 2019). Since the dosage of herbal medicines and duration of treatment can affect menstrual regularity, the interventions longer than 3 months might have different results. 

In this study, hormonal levels had no significant difference between groups after the intervention, except DHEAS and TSH levels in Ferula vs. placebo group. One animal study found that Ferula resin can decrease the LH to FSH ratio in PCOS rat (Morovatisharifabad et al., 2020) Use of Urtica dioica in hyper-androgenic women can regulate testosterone levels (Najafipour et al., 2014). Also, use of Genistein can significantly improve serum levels of LH, triglyceride, low density lipoprotein cholesterol, DHEAS and testosterone after 3 months (Khani et al., 2011). Three months Vitex therapy can decrease prolactin level in women with complaints of cyclic mastalgia (Dinç and Coşkun, 2014). Low et al. concluded that phytoestrogens modulate sex hormones and SHBG levels in postmenopausal women (Low et al., 2005).

In the placebo group, significant decreases in terms of free testosterone, FSH level, and ovarian volumes after 3 months were seen. Although confounding factors such as unstable follicular phase in women with oligomenorrhea and the un- exact nature of trans-abdominal ultrasound measurements might be related to these results in the placebo group.

In the current trial, ovarian follicular numbers significantly decreased within the Ferula and Fennel groups after treatment but, ovarian volumes did not change in both groups. There is no study which evaluated the effect of herbal medicine on ovarian volume, but the use of metformin (Sanoee et al., 2011) and Trigonella foenum-graecum can decrease both ovarian volumes in PCOS women (Swaroop et al., 2015). Different results in terms of ovarian volume and number of follicles in different studies could be due to the physiology of the ovarian tissue.

Limitations of this study were as follows: small sample size, poor generalizability and the short duration of treatment. For future studies, measurement of ovarian cysts by trans-vaginal ultrasound after herbal medicine therapy is recommended.

In conclusion, since Ferula has been able to cause significant changes in TSH and DEHAS hormones and reduce the number of right and left ovarian follicles compared to Fennel and placebo, this herbal medicine is more effective than Fennel in managing PCOS.

## Conflicts of interest

The authors have declared that there was not any financial or technical relationship with Barij Essence Company in the present study.
